# Wastewater Surveillance of Aichi Virus in Baltimore

**DOI:** 10.3390/pathogens15070728

**Published:** 2026-07-10

**Authors:** Daniel A. Nwaubani, Rakshya Baral, Tamunobelema Solomon, Mustafa Ali, Tania Moharrery, Samendra P. Sherchan

**Affiliations:** 1 Center of Research Excellence in Wastewater Based Epidemiology, Morgan State University, Baltimore, MD 21251, USA; 2 Bioenvironmental Science Program, Morgan State University, Baltimore, MD 21251, USA

**Keywords:** Aichi virus, wastewater-based epidemiology, Baltimore, fecal contamination indicators, RT-qPCR, enteric viruses, physicochemical parameters

## Abstract

This study established long-term wastewater surveillance of Aichi virus (AiV) in Maryland. AiV, a member of the Kobuvirus genus associated with acute gastroenteritis, has established itself as an integral marker for wastewater-based monitoring; however, two key research questions remain unaddressed for the Baltimore metropolitan area: (1) whether AiV is consistently detectable in municipal wastewater throughout the year, and (2) whether its concentrations exhibit a measurable seasonal pattern. To address these hypotheses, influent samples were collected on a weekly basis from WWTP-A and WWTP-B from January to December 2023 (with grab sampling conducted at WWTP-A and automated collection deployed for the influent sampling of the water treatment plant B). All samples (*n* = 51) were subjected to PEG 8000 concentration, RNA extraction, cDNA synthesis, and RT-qPCR quantification. We observed AiV RNA in 94.12% of the samples from both facilities (25/51 at WWTP-A and 23/51 at WWTP-B) with concentrations that ranged from 2.5 to 3.63 log_10_ gc/L and a seasonal pattern showing consistent declines: loads for WWTP-A declining from winter (3.58 log_10_ gc/L) to fall (2.56) and for WWTP-B from winter (3.28 log_10_ gc/L) to fall (2.31). The year-round constant AiV presence provides a strong basis for its use as a stable viral marker within wastewater-based epidemiology efforts.

## 1. Introduction

Wastewater-based epidemiology (WBE) emerged as a critical tool for tracking viral pathogens at the community scale. It applies to the concept that viruses shed in human stool or urine can be detected in municipal sewage, providing a noninvasive, cost-effective way to track community health threats and disease outbreaks. During the COVID-19 pandemic [1], wastewater surveillance proved essential for tracking community-level infection dynamics and informing public health responses nationally and worldwide [2]. Wastewater-based epidemiology (WBE) has been widely used to detect pathogens such as poliovirus, norovirus, hepatitis A, Mpox virus, and SARS-CoV-2, with Baltimore, Maryland actively participating in such surveillance efforts. Notably, Sherchan et al. demonstrated that Mpox virus DNA can be effectively detected and monitored in untreated wastewater, providing valuable insights into community-level prevalence to guide public health interventions [3].

Although WBE has been widely applied to viral pathogens, surveillance of the Aichi virus (AiV) in urban settings such as Baltimore remains limited. AiV belongs to the Kobuvirus genus and is found mainly in acute gastroenteritis. Its detection in wastewater samples indicates its public health significance and the need for systematic surveillance. In wastewater samples worldwide, AiV has consistently been detected. One seminal Dutch study detected AiV RNA in 100% of analyzed sewage samples, suggesting its widespread circulation in human populations [4]. Kitajima et al. reported the detection of AiV in influent and effluent wastewater at two treatment plants in southern Arizona, United States, where AiV was detected in 100% of samples and showed greater abundance and lower treatment reduction than other human enteric viruses [5]. High detection rates have also been reported from Brazil, Japan, and other countries, confirming that AiV is an environmental contaminant and a public health problem worldwide [6]. AiV shows resistance to wastewater treatment processes. do Nascimento et al. reported long-term persistence of AiV in sewage, with a 90.4% detection rate in untreated sewage, and only slight reductions following treatment 78.8% after anaerobic biological treatment and 71.1% after chemical treatment, underscoring AiV’s resilience in wastewater environments [7]. do Nascimento et al. demonstrated that RT-qPCR (and duplex RT-LAMP) can reliably detect AiV A in untreated wastewater samples, showing the method’s effectiveness in environmental water monitoring [8]. Meanwhile, metagenomic next-generation sequencing approaches enable simultaneous detection and genotyping of multiple viral pathogens in wastewater, providing a broader epidemiological picture [9].

Physicochemical characteristics of wastewater, such as temperature, pH, salinity, electrical conductivity (EC), and ionic strength, have been reported to affect survival, decay, and transport of enteric viruses in water bodies [10]. One of the most significant covariates is temperature, where higher temperatures lead to an accelerated viral inactivation through breakdown of capsid proteins and genomic RNA, as has been shown with several enteric viruses [11,12]. Viral persistence can be influenced by pH, where alkaline conditions (high pH) promote capsid disassembly or destabilization in many non-enveloped viruses, thereby reducing their environmental survivability [13,14]. Ionic strength and salinity also impact virus particle interactions: at high salt concentrations, viral adsorption on solids or on the filtration surface can increase, causing a decrease in virions that are freely suspended [15]. Although these pathways have been described for other enteric viruses, in the case of AiV, only scarce evidence could be provided. AiV has been reported to be remarkably stable in sewage and surface waters [4], but the impact of physicochemical variables on this resilience is unknown. Understanding these relationships is necessary to accurately interpret AiV concentration signals in wastewater and to distinguish population-level fecal viral excretion patterns from environmentally driven variation in observed concentrations. With an existing wastewater surveillance infrastructure in Baltimore, monitoring of AiVs can be introduced in a timely manner. Baltimore was selected due to its established wastewater surveillance infrastructure from Maryland’s pioneering COVID-19 Sewer Sentinel Initiative [16,17], federal consent decree oversight with over $1 billion in infrastructure investments [18], and operation under the nation’s most stringent nutrient removal standards for Chesapeake Bay protection [19,20], providing an ideal regulated environment with proven surveillance protocols for establishing AiV monitoring baselines. As a result, active AiV surveillance would make it possible to detect early and respond promptly to outbreaks. Surveillance data would also provide useful epidemiological information on the prevalence, seasonality, and community exposure patterns of AiV in Baltimore.

## 2. Materials and Methods

### 2.1. Wastewater Sample Collection at Two Baltimore WWTPs

For this study, two wastewater treatment plants in Baltimore, designated WWTP-A and WWTP-B, were selected from among Maryland’s facilities. WWTP-A is a two-stage wastewater treatment plant with a capacity of 73 million gallons per day (approximately 276,300 m^3^/day) MGD and serves about 1.3 million people across Baltimore City and County (approximately 492,100 m^3^/day). Raw wastewater—a mix of household sewage, agricultural runoff, and industrial discharge—enters the plant and is treated through secondary and tertiary stages. After dechlorination, 40–50% of the treated water is reused for industrial purposes, while the rest is discharged into a nearby river. Surveillance of PI with specimens received at these facilities gives a window on the spread of AiV in Baltimore.

Between 24 January 2023 and 5 December 2023, weekly primary influent (PI) samples (*n* = 51) were collected from both wastewater treatment plants. At WWTP-A, a 1 L grab sample was collected directly from the raw wastewater influent inlet. At WWTP-B, a 1 L composite sample was collected using an automated sampler programmed to obtain 200 mL of influent wastewater every 30 min over a 24 h period. All PI samples were collected between 06:00 and 10:00 h in 1 L clear high-density polyethylene (HDPE) bottles, maintained at 4 °C during transport, and processed in the laboratory immediately upon arrival each week.

### 2.2. Concentration and RNA Extraction

45 mL of each PI sample was transferred into 50 mL centrifuge tubes and centrifuged at 4700× *g* for 30 min at 4 °C (Sigma-Aldrich, Darmstadt, Germany) to pellet the sediments and large debris. Subsequently, the supernatant was transferred to a fresh tube with 4.0 g PEG 8000 (Sigma-Aldrich, St. Louis, MO, USA) and 0.94 g NaCl (Sigma-Aldrich, St. Louis, MO, USA) by hand shaking, followed by vortexing for an additional 10 min. The viral particles were pelleted at 12,000× *g* for 99 min. The supernatant was discarded, leaving approximately 5 mL in each tube. The tubes were centrifuged again at 12,000× *g* for 5 min at 4 °C to recover residual virus from the tube walls. The final pellet was resuspended in 600 µL PCR-grade water, vortexed, briefly centrifuged, and stored at –20 °C for 24 h. RNA was extracted/purified from this concentrate using QIAamp Viral RNA Mini Kit (QIAGEN, Hilden, Germany)) as per the manufacturer’s protocol [3].

### 2.3. RT-qPCR Assay

Five microliters of extracted RNA were converted into a 10 µL complementary DNA (cDNA) reaction using the High-Capacity cDNA Reverse Transcription Kit with RNase inhibitor (Applied Biosystems™, Thermo Fisher Scientific, Waltham, MA, USA), following the manufacturer’s protocol. The resulting cDNA was analyzed for the viral target using a Bio-Rad Opus CFX96 real-time RT-qPCR system (Bio-Rad Laboratories, Hercules, CA, USA).

RT-qPCR assays for AiV were performed using a CFX96 real-time PCR instrument (Bio-Rad Laboratories, Hercules, CA, USA). Each 25 μL reaction mixture contained 12.5 μL of qPCR ToughMix (Quantabio, Beverly, MA, USA), 2 μL of forward primer (10 µM final concentration), 2 μL of reverse primer (10 µM final concentration), 1.5 μL of probe (5 µM final concentration), and 6.5 μL of molecular-grade water. Each reaction received 2.5 μL of cDNA template. The primer and probe sequences used were those described by Kitajima et al. for universal detection of AiV (Supplementary Table S1: Primer and Probe Sequences for Aichi Virus (AiV) qPCR) [21].

### 2.4. Measurement of Wastewater Samples Physical Parameters

Physical parameters, including pH, total dissolved solids (TDS) (ppt), electrical conductivity (µS/cm), salinity (ppm), and temperature (°C) were measured using an Oaklon PCTSTestr™ 50 (5-in-1) water analysis instrument (Cole-Parmer, Vernon Hills, IL, USA).

## 3. Results

### 3.1. Detection of AiV in Wastewater Samples

**Figure 1:**
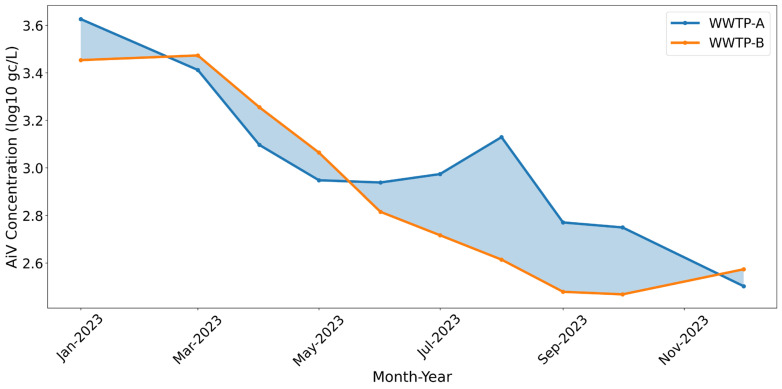
Monthly Aichi virus (AiV) concentrations (log_10_ gc/L) detected in influent wastewater at WWTP-A and WWTP-B during 2023.

The average monthly log_10_ AiV genomic copies per liter (gc/L) in influent sewage at WWTP-A and WWTP-B from January to December 2023 are shown in Figure 1. Values represent 3-point rolling monthly means calculated from all available sampling events. The shaded region highlights the temporal divergence between the two plants, indicating consistently higher AiV loads at WWTP-A during the first half of the year. Both plants exhibited a gradual decline in AiV concentrations toward late 2023.

Overall, AiV levels demonstrated a steady decline across the year, beginning at 3.63 log_10_ gc/L in January at WWTP-A and falling to approximately 2.5–2.6 log_10_ gc/L by late autumn. These trends reflect temporal variability in AiV presence within the respective sewersheds and demonstrate the utility of wastewater-based epidemiology for monitoring enteric viral dynamics.

This pattern indicates moderate intra-annual variation rather than complete stability.

### 3.2. Seasonal Variation in AiV Virus in Wastewater Samples

**Figure 2:**
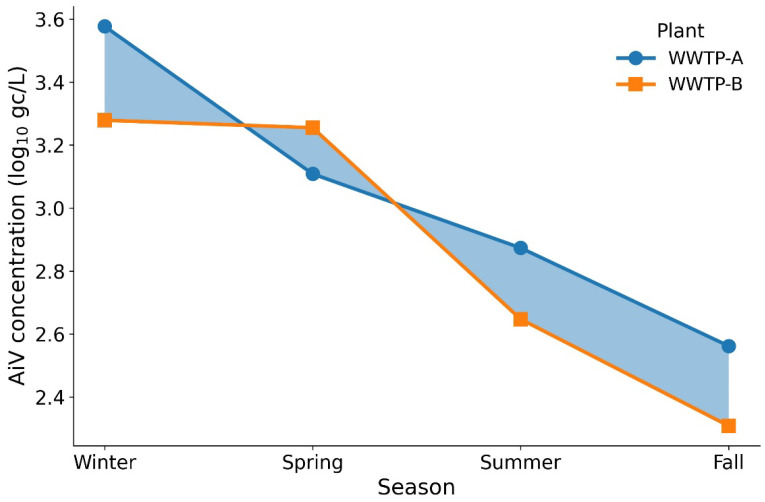
Seasonal variation in AiV virus concentrations (log_10_ gc/L) in influent wastewater from WWTPA and WWTPB in Maryland, USA.

Figure 2 illustrates the seasonal variation in Aichi virus (AiV) concentrations measured in influent wastewater from WWTP-A and WWTP-B during 2023. Seasons were defined as winter (December–February), spring (March–May), summer (June–August), and fall (September–November). Lines represent mean seasonal concentrations, and the shaded ribbon indicates differences between treatment plants.

The shaded ribbon in Figure 2 visually highlights the magnitude of seasonal separation between the two plants, with WWTP-A consistently exhibiting higher AiV concentrations than WWTP-B across all seasons, typically by 0.20 to 0.30 log units, a difference larger than analytical noise. Both plants show their highest concentrations in winter and a progressive decline into spring, summer, and fall, suggesting a modest but measurable seasonal influence on AiV loads in wastewater.

### 3.3. Correlation Between Virus Concentration and Physicochemical Parameters

At WWTP-A, AiV concentrations showed weak negative correlations with all physicochemical parameters (pH: −0.27, conductivity: −0.33, TDS: −0.33, salinity: −0.22, temperature: −0.32), while conductivity and TDS were strongly intercorrelated (r = 0.97). At WWTP-B, correlations between AiV and the same parameters were generally weak and mixed in direction (range: −0.15 to 0.23), with no strong intercorrelations observed among physicochemical variables.

## 4. Discussion

At WWTP-A (Figure 1), concentrations started at 3.63 log_10_ gc/L in January, decreased slightly to ~3.55 in February, and continued downward to ~3.40 in March. Levels dropped further through April (~3.10) and May (~2.95). A period of mild stabilization occurred in June (~2.94) and July (~2.98), followed by a minor rebound in August (~3.11 log_10_ gc/L). After this point, concentrations declined steadily into September (~2.77), October (~2.75), and reached their lowest value in November (~2.53 log_10_ gc/L). The curve suggests a smooth, season-linked decline with only small short-term fluctuations.

At WWTP-B (Figure 1), concentrations followed a similar seasonal trajectory but at slightly lower levels. Values began near 3.45 log_10_ gc/L in January–February and remained around ~3.46–3.47 through March. A gradual decrease continued into April (~3.25) and May (~3.07). A more pronounced decline was evident from June (~2.82) through July (~2.72) and August (~2.60 log_10_ gc/L). After reaching this minimum, concentrations stabilized in September (~2.51) and October (~2.50), with a slight increase toward November (~2.57 log_10_ gc/L).

The shaded region between the two curves reflects a modest but consistent divergence between the plants, with WWTP-A typically reporting concentrations 0.05–0.15 log_10_ gc/L higher than WWTP-B for most months. Despite this difference, their temporal trends remained highly parallel, demonstrating similar environmental influences, population shedding patterns, and treatment system stability at both facilities.

The AiV concentrations measured in this study (≈2.5–3.63 log_10_ gc/L) fall within the range reported in previous wastewater studies from other settings. For example, Kitajima et al. reported influent concentrations of 10^3^–10^5^ copies/L (≈3–5 log_10_), which overlaps with the upper concentrations observed at the Baltimore WWTPs [22]. Likewise, do Nascimento et al. detected AiV in raw sewage at 2.8 × 10^3^–6.3 × 10^4^ copies/L (≈3.45–4.80 log_10_) in a Brazilian WWTP [7].

**Table 1:** Comparative Analysis of Aichi virus (AiV-1) detection in wastewater/sewage and wastewater-contaminated shellfish.

Country/Region	City/Location	Sample Type	Study Design	Detection Rate/Key Result	Reference
United States (Arizona)	Southern Arizona (municipal WWTP; city not specified)	Raw & treated municipal wastewater	Monthly over 13 months; 13 influent + 13 effluent	100% detection in influent and effluent; up to ~10^5^ gc/L; genotype B dominant	[23]
Japan	Multiple WWTPs and rivers (cities not specified)	WWTP influent & effluent; river water	Influent *n* = 12; effluent *n* = 12; river *n* = 60	Influent 100%; effluent 92%; river 60%	[22]
Japan	Same WWTPs as above (cities not specified)	WWTP influent & effluent	Monthly for 1 year; 12 influent + 12 effluent	Up to 2.2 × 10^7^ copies/L (influent); 1.8 × 10^4^ copies/L (effluent)	[21]
Netherlands	National surveillance (RIVM; cities not specified)	Archival sewage; surface water	1987–2000 & 2009–2012; sewage *n* = 8/period	Sewage 100%; surface water 71–100%	[4]
Italy	Province of Teramo (Central Italy)	Untreated influent sewage	48 influent samples	6/48 (12.5%) positive	[24]
Tunisia	Monastir region	Sewage & shellfish	Environmental surveillance	Sewage 6%; shellfish 6.6%	[25]
Venezuela	Urban sewage-polluted river (Caracas)	River water	*n* = 11	5/11 (45%) positive; genotype B	[26]
Senegal	Dakar (Cambérène WWTP)	Raw, decanted & treated wastewater	Raw *n* = 20; decanted *n* = 19; treated *n* = 27	Raw 70%; decanted 68.4%; treated 59.3%	[27]
Uruguay	Bella Unión, Salto, Paysandú, Fray Bentos	Municipal wastewater	Biweekly March 2011–February 2012; *n* = 96	54/96 positive; higher in colder months	[28]
Thailand	Chiang Mai city	Environmental waters incl. wastewater	Monthly November 2016–July 2018; *n* = 126	Overall 22.2%; wastewater 52.4%	[29]
China	Eastern China (Jinan)	Raw municipal sewage	Monthly surveillance 2019–2022; *n* = 48	95.83% (46/48) positive; up to ~2.7 × 10^5^ gc/L	[30]
Brazil	São José do Rio Preto, São Paulo State	Raw sewage and treated stages	One-year surveillance; *n* = 156	90.4% raw; 78.8% post-anaerobic; 71.1% post-chemical treatment	[7]

Overall, comparison with global data demonstrates that the Baltimore findings are not anomalous but instead reflect a broader, well-documented pattern of widespread AiV presence and persistence in wastewater systems. The combination of high detection frequency, moderate and stable concentrations, and limited seasonal variability further supports the utility of AiV as a robust viral marker for fecal contamination and WBE in urban environments.

The detection rate described in this study has similar patterns to those reported in other high-prevalence populations. For example, a 1-year sewerage monitoring program in southeastern Brazil demonstrated detection of AiV-A in 90.4% raw sewage, 78.8% post-anaerobic treatment, and 71.1% post-chemical treatment samples, highlighting the virus’s abundant presence in untreated influent and persistence through wastewater processing [7]. Studies based on areas with lower detection rates further contextualize the phenomenon. In Egypt, Shaheen et al. reported AiV positivity in 22% of raw sewage samples, 14% of treated effluent, and even up to 18% in environmental water bodies, showing that prevalence can vary considerably depending on geographical, environmental, and methodological aspects [31].

High detection frequencies (nearly persistent or fully persistent presence have been recorded in some long-term monitoring schemes. In Japan, Kitajima et al. observed detection of AiV in all influent and 92% effluent wastewater samples during several sampling rounds [22], whereas Lodder et al. detected AiV RNA in 100% of Dutch sewage over a multi-decadal recording period [4]. While rates here are higher than the 94.12% at Baltimore, they are within anticipated global levels and confirm AiV as endemic in human wastewater. The Baltimore findings are within the upper range of global detection frequencies (∼60–100%) and indicate that AiV is an endemic, commonly excreted enteric virus in urban populations.

The year-round detection of AiV at both treatment plants, with relatively stable concentrations (~2.5–3.6 log_10_ gc/L), suggests that AiV circulates endemically in the Baltimore population and is continuously shed into wastewater at consistent levels. This stable pattern supports the use of AiV as a reliable fecal viral indicator in wastewater-based epidemiology.

The high detection rate of AiV in the present study (94.12% positive prevalence in influent) is within an upper boundary of that reported for municipal wastewater treatment plants worldwide (Table 1). AiV occurrence in influent sewage appears nearly ubiquitous based on long-term surveillance studies in Japan and the Netherlands, where detection frequencies ranged from 92% to 100%, indicating that AiV presence is common and persistent within human populations.

Also, high prevalence has been reported in recent studies from China (95.83%) and Brazil (90.4% in raw sewage), supporting the view that AiV is a prevalent and stable component of domestic sewage in populated urban areas.

Conversely, greatly reduced detection rates have been measured in a number of geographic locations, such as Italy (12.5%) and Thailand (22.2%), as well as moderate prevalence in Senegal (70% by influent raw) and Uruguay (56%). These differences likely represent variation in population shedding kinetics, climatic conditions, wastewater infrastructure, sample size, and analytical methodologies rather than lack of virus circulation. Together, the literature indicates that AiV prevalence in wastewater varies widely on a global scale (~10–100%); Baltimore falls within this range of high-prevalence settings.

Measured AiV concentrations in Baltimore influent wastewater (approximately 2.5–3.63 log_10_ gc/L) also fall well within the range reported internationally. Comparable concentration levels have been described (Table 1) in Japan (up to 10^7^ gc/L in influent), China (10^3^–10^5^ gc/L), Brazil (10^3^–10^4^ gc/L), and Australia (3.6–6.2 log_10_ gc/L) [8]. Although absolute concentrations vary across studies, the relatively narrow and stable concentration range observed in Baltimore supports the interpretation that AiV exhibits limited temporal variability and strong environmental persistence in sewage matrices.

In Figure 2, the seasonal means show a clear decreasing pattern from winter through fall. At WWTP-A, concentrations declined from 3.58 log_10_ gc/L in winter to 3.11 log_10_ gc/L in spring, 2.87 log_10_ gc/L in summer, and 2.56 log_10_ gc/L in fall. A similar but slightly lower pattern was observed at WWTP-B, with seasonal means of 3.28 log_10_ gc/L (winter), 3.25 log_10_ gc/L (spring), 2.65 log_10_ gc/L (summer), and 2.31 log_10_ gc/L (fall).

These results are in agreement with earlier studies that had recorded yearlong presence, with few, albeit noticeable seasonal variations. Chen et al. reported persistence of AiV in Chinese wastewater between 2019 and 2022 with levels ranging between 3.1 and 5.4 log_10_ gc/L and observed only slight season dependency [30]. Likewise, do Nascimento et al. in Brazilian sewage detected the AiV-A between 2.05 and 4.72 log_10_ gc/L without significant differences between rainy and dry seasons [7]. Previous research engaging in such surveillance was likewise found not to have distinct seasonal peaks, as was the case of Kitajima and Gerba from Japan [32]. Taken together, our observations indicate that the Aichi virus is endemic in the human population and remains in wastewater all year round.

The consistent detection and gradual seasonal decline observed in this study further underscore AiV’s environmental stability and support its use as a baseline fecal contamination indicator for benchmarking removal efficiencies and interpreting the behavior of other enteric viruses in wastewater systems. WWTP-A samples were collected using grab sampling, whereas WWTP-B samples were obtained using an automated composite sampling approach. Therefore, observed differences between the two plants should be interpreted primarily as inter-plant variability, while recognizing that differences in sampling strategy may also contribute to the measured concentrations.

**Figure 3:**
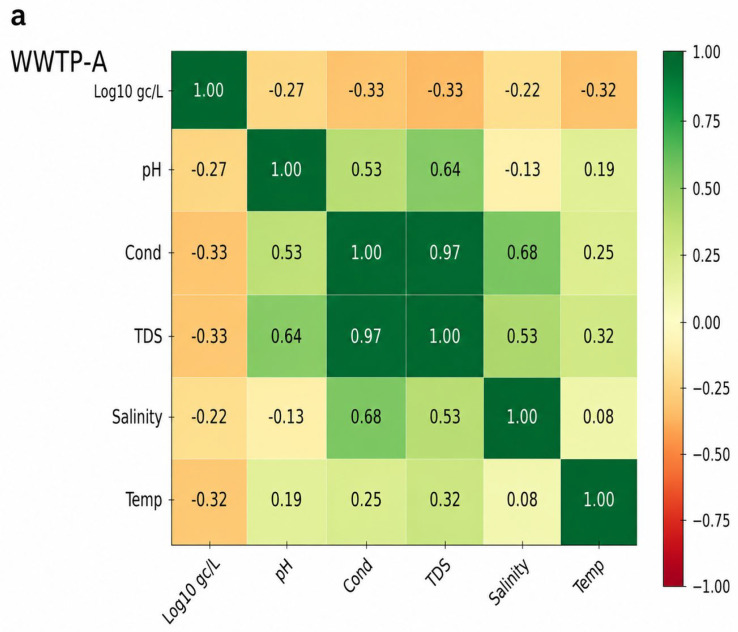

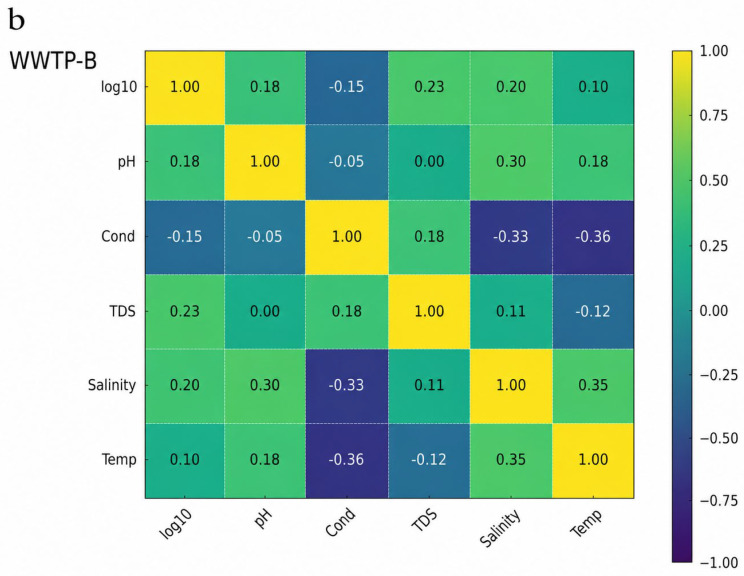
Correlation heatmaps showing relationships between AiV virus concentration and physicochemical parameters at WWTP-A (**a**) and WWTP-B (**b**).

For WWTP-A, the correlation statistics (Figure 3a) all showed statistically non-significant poor negative correlation with AiV concentrations, including pH, conductivity, TDS, and temperature. All Pearson correlation coefficients were approximately between –0.22 and –0.33, and none were statistically significant (*p* > 0.05). This trend suggested that viral concentrations were slightly lower with increased ionic strength, salinity, or temperature, but the differences were limited.

The weak inverse associations with pH and temperature indicated that AiV signals were likely to decrease in more alkaline and warmer environments. This finding is consistent with previous evidence that higher temperatures and pH values facilitate a rapid loss of viral capsid integrity and RNA stability, thereby leading to decreased persistence in the environment [12]. Similarly, the weak negative associations with conductivity, TDS, and salinity suggest that an increase in ionic strength does not stabilize AiV more strongly in this wastewater system and could even be partly responsible for viral decay or removal.

These relationships are illustrated in Supplementary Figure S1 (scatterplots showing relationships between Aichi virus concentration and individual physicochemical parameters at WWTP-A), where regression lines show slight downward trends with increasing values of the measured physicochemical parameters. All the relatively low correlation coefficients (|r| < 0.33) indicated that wastewater chemistry is less likely to be involved in AiV abundance at WWTP-A. Rather, viral concentrations are better related to fecal viral excretion upstream, hydraulic retention time, flow fluctuation, and performance of the treatment system.

Additionally, the identification of the Aichi virus in sewage has further ecological and public health significance. Bioaccumulation of AiV in shellfish and the marine environment has been well reported, with links to gastroenteritis outbreaks in France [33] and finding positive coastal shellfish samples from Tunisia [25].

Figure 3b shows the correlation matrix obtained between AiV concentration and physicochemical parameters measured for influent wastewater at WWTP-B. Taken together, there were predominantly weak relationships with correlation coefficients from 0.05 to 0.52, indicating negligible chemical control on viral levels. Of all the factors considered, temperature was found to have a statistically significant and strongest correlation (r ≈ 0.52, *p* < 0.01), with positive cases of AiV being more frequently detected when it was warm. This implies there may be greater sensitivity of viral detection to seasonal fecal viral excretion patterns or influent dilution than decay directly through thermal inactivation.

On the other hand, pH showed a weak positive and non-significant correlation (r ≈ 0.34) with salinity, conductivity, and TDS also showing weak values of associations between them (r ≈ 0.05–0.20). These weak correlations indicate that these parameters operate within similar ranges at WWTP-B, and the contribution of ionic strength to the general chemistry of wastewater did not appreciably impact AiV persistence or detectability.

These relationships appear to be stronger in Supplementary Figure S2 (Relationships between Aichi virus concentration and individual physicochemical parameters at wastewater treatment plant B), as indicated by slow regression trends and strongly grouped data points for all parameters other than temperature. The temperature scatterplot has a small positive trend due to its small and fully positive correlation with viral concentration, while all other feature peaking values show no trend or near-zero slope against viral load. This indicates that the abundance of Aichi virus in WWTP-B is determined mainly by upstream human contributions and flow dynamics, rather than the physicochemical properties of the wastewater matrix.

Taken together, Figure 3b and Supplementary Figure S2 (Relationships between Aichi virus concentration and individual physicochemical parameters at wastewater treatment plant B) indicate that physicochemical factors at WWTP-B exert minimal influence on AiV behavior, while reinforcing the virus’s robustness as a stable and reliable indicator of human fecal contamination in wastewater-based epidemiology.

## 5. Conclusions

This study established the first comprehensive year-long surveillance of Aichi virus (AiV) in Baltimore wastewater and demonstrated its remarkable feasibility as a stable marker for wastewater-based epidemiology programs. An exceptionally high frequency (94.12%) of detection across both major treatment plants verifies the consistent existence of AiV in urban wastewater under all seasons, establishing it as a very stable biomarker that can be used for virus monitoring at the community level.

The seasonal dynamics we observed, in which AiV concentrations dropped from winter peaks (3.58 log_10_ gc/L for WWTP-A; 3.28 log_10_ gc/L for WWTP-B) during winter months down to fall minimums (2.56 and 2.13/log_10_/gc/L, respectively), will serve as important baseline data going forward that can be used to inform future surveillance programs. This detection, regardless of the methodology used for sampling both facilities, solidifies the robustness for AiV as a target for surveillance.

These results advocate the incorporation of AiV monitoring policies in municipal WBE programs as a complementary viral target alongside classical viral determinants. Safe detection rates and predictable seasonal patterns, combined, make AiV especially valuable for long-term epidemiological studies and community health assessments in such urban settings as Baltimore. For Aichi virus (AiV), we recommend that future research efforts correlate AiV wastewater concentrations with clinical gastroenteritis data to determine qualitative relationships between environmental detection and community disease burden.

Future studies should investigate AiV behavior in treated effluent and downstream environments to better understand its environmental fate and public health implications.

## Data Availability

The original contributions presented in this study are included in the article/Supplementary Material. Further inquiries can be directed to the corresponding author(s).

## Supplementary Materials

The following supporting information can be downloaded at https://www.mdpi.com/article/10.3390/pathogens15070728/s1, Figure S1: Scatterplots showing relationships between Aichi virus concentration and individual physicochemical parameters at WWTP-A; Figure S2: Scatterplots showing relationships between Aichi virus concentration and individual physicochemical parameters at WWTP-B; Table S1: Primer and Probe Sequences for Aichi Virus (AiV) qPCR.

## Abbreviations

The following abbreviations are used in the manuscript:

AiV	Aichi virus
WWTP	Wastewater Treatment Plant
PEG	Polyethylene Glycol
cDNA	complementary DNA
RT-qPCR	Reverse Transcription Quantitative Polymerase Chain Reaction
gc/L	Genome Copies per Liter
WBE	Wastewater-based epidemiology
COVID-19	Coronavirus Disease 2019
SARS-CoV-2	Severe Acute Respiratory Syndrome Coronavirus 2
RNA	Ribonucleic Acid
RT-LAMP	Reverse Transcription Loop-Mediated Isothermal Amplification
EC	electrical conductivity
MGD	Million Gallons per Day
PI	Primary Influent
TDS	Total Dissolved Solids
pH	potential of hydrogen
