# Supplementary material for: Wastewater Surveillance of Aichi Virus in Baltimore
Source: Pathogens. 2026 Jul 10;15(7):728. doi: 10.3390/pathogens15070728 (PMC13414499; doi:10.3390/pathogens15070728)
Supplement: Supplementary file 1 [file pathogens-15-00728-s001.zip › pathogens-4204641-supplementary.pdf]

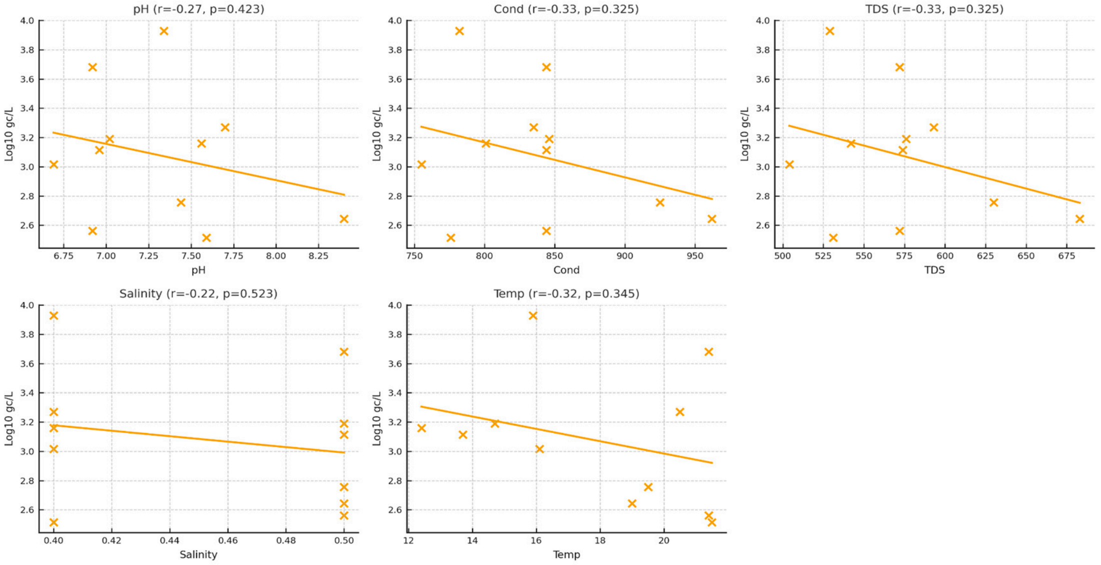

Supplementary Figure S1. Scatterplots showing relationships between Aichi virus concentration and individual physicochemical parameters at WWTP-A

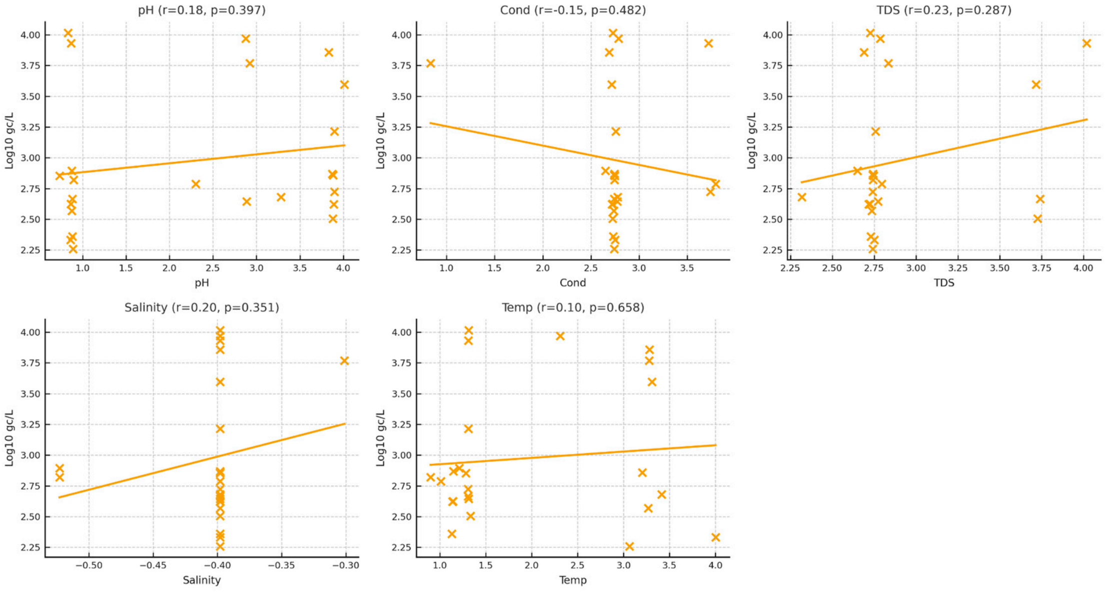

Supplementary Figure S2. Scatterplots showing relationships between Aichi virus concentration and individual physicochemical parameters at WWTP-B

Supplementary Table S1. Primer and Probe Sequences for Aichi Virus (AiV) qPCR

Primer/Probe Type	Name	Sequence (5′ → 3′)
Forward primer	AiV-AB-F	GTCTCCACHGACACYAAYTGGAC
Reverse primer	AiV-AB-R	GTTGTACATRGCAGCCCAGG
Probe	AiV-AB-TP	FAM-TTYTCCTTYGTGCGTGC-MGB-NFQ
